# Quantitative Analysis of Diagnostic Reasoning Using Initial Electronic Medical Records

**DOI:** 10.3390/diagnostics15121561

**Published:** 2025-06-18

**Authors:** Shinya Takeuchi, Yoshiyasu Okuhara, Yutaka Hatakeyama

**Affiliations:** 1 Department of Disaster and Emergency Medicine, Kochi Medical School, Kochi University, Nankoku 783-8505, Kochi, Japan; 2 Centre of Medical Information Science, Kochi Medical School, Kochi University, Nankoku 783-8505, Kochi, Japan; okuharay@kochi-u.ac.jp (Y.O.); hatake@kochi-u.ac.jp (Y.H.)

**Keywords:** diagnostic reasoning, electronic medical records, clinical reasoning, natural language processing, medical education, text analysis

## Abstract

**Background/Objectives**: Diagnostic reasoning is essential in clinical practice and medical education, yet it often becomes an automated process, making its cognitive mechanisms less visible. Despite the widespread use of electronic medical records, few studies have quantitatively evaluated how clinicians’ reasoning is documented in real-world electronic medical records. This study aimed to investigate whether initial electronic medical records contain valuable information for diagnostic reasoning and assess the feasibility of using text analysis and logistic regression to make this reasoning process visible. **Methods**: We conducted a retrospective analysis of initial electronic medical records at Kochi University Hospital between 2008 and 2022. Two patient cohorts presenting with dizziness and headaches were analysed. Text analysis was performed using GiNZA, a Japanese natural language processing library, and logistic regression analyses were conducted to identify associations with final diagnoses. **Results**: We identified 1277 dizziness cases, of which 248 were analysed, revealing 48 significant diagnostic terms. Moreover, we identified 1904 headache cases, of which 616 were analysed, revealing 46 significant diagnostic terms. The logistic regression analysis demonstrated that the presence of specific terms, as well as whether they were expressed affirmatively or negatively, was significantly associated with diagnostic outcomes. **Conclusions**: Initial EMRs contain quantifiable linguistic cues relevant to diagnostic reasoning. Even simple analytical methods can reveal reasoning patterns, offering valuable insights for medical education and supporting the development of explainable diagnostic support systems.

## 1. Introduction

The thought process that clinicians use to identify a patient’s disease is called diagnostic reasoning, which is essential for all physicians to function efficiently and fulfil their roles [1]. Diagnostic reasoning is also included in the medical education curricula [2,3]. Many elements of diagnostic reasoning have been identified, represented by probabilistic, causal and deterministic reasoning [4]. Probabilistic reasoning is used to formulate diagnostic hypotheses and relies on statistical relationships between keywords. Causal reasoning examines whether the diagnostic hypothesis is reasonable and consistent in terms of causal relationships. However, causal reasoning is not useful for forming hypotheses. Deterministic reasoning is the routine examination or procedure for well-encountered problems. This cannot be used successfully without knowledge and experience. Physical findings and medical history are needed to provide evidence for all elements of diagnostic reasoning. Although diagnostic reasoning is conducted daily in clinical practice, it becomes automated owing to experience, causing it to be recognised less consciously. The validity of existing clinical reasoning education can be ensured by quantitatively verifying diagnostic reasoning with clear evidence and making it logically reproducible. This is expected to significantly enhance the effectiveness and quality of future medical education and facilitate the development of computer-assisted diagnostic support systems that explain their reasoning.

With the widespread adoption of electronic medical records (EMRs) and the accumulation of electronic medical data, the potential to logically formalise the diagnostic reasoning processes that physicians undertake in diagnosing and selecting treatment is growing [5,6]. This can be achieved using large-scale clinical data in a comprehensible manner. However, such attempts have been scarce. Furthermore, most studies on diagnostic reasoning using large-scale clinical data have employed machine learning techniques that are difficult for humans to interpret, with mixed impacts on clinical reasoning performance [7,8,9,10]. For instance, Shen et al. used Naive Bayes to correct symptom correlations rather than assuming complete independence and extracted symptom-disease knowledge triplets from entire EMRs [11]. However, the objective of this study—to make the reasoning process visible—differs fundamentally from that of previous research, which primarily aimed to ensure the accuracy of diagnostic classification. Therefore, the methodologies used in prior studies are not directly applicable to the goals of the present study. Although these approaches hold the potential for implementation as clinical support tools, they fail to serve educational roles, such as explaining diagnoses to patients or transferring knowledge and experience to medical students and junior physicians.

Effective patient records should document clinicians’ thoughts concerning patients and their problems [12]. However, even after several decades of implementation, our understanding of concepts and relationships in diagnostic reasoning remains insufficient [13]. Only three studies have directly assessed the methods that clinicians use to interpret clinical cases and record their reasoning in EMRs [13,14,15]. Farri et al. used the think-aloud protocol to observe primary care physicians reviewing cases using the EMRs and developed cognitive pathways for clinicians to read/search medical records and to assess and plan [15]. As a prerequisite, it is necessary to understand how EMRs are currently used to support clinical reasoning and documentation. In addition, all these studies evaluated simulated outpatient visit notes. To the best of our knowledge, no studies have assessed real-world data. Initial clinical records in real-world settings contain a wealth of information useful for diagnostic reasoning, such as chief complaints, present illness, past medical history, family history, and physical examinations. However, the free text and unstructured nature of initial EMRs pose challenges for secondary use [16]. Whether these records contain useful information for diagnostic reasoning remains unclear. Therefore, this study aimed to investigate whether initial EMRs contain information valuable for diagnostic reasoning and assess the feasibility of implementing a visible reasoning system based on these records.

## 2. Methods

This study used EMR system data from Kochi University Hospital in Kochi Prefecture, Japan. The target data were initial EMRs between 2008 and 2022. We extracted two cohorts for analysis: patients presenting with dizziness and those presenting with headaches. These chief complaints were selected due to their prevalence, ease of securing an adequate number of cases, and the necessity of ruling out critical conditions.

The first cohort comprised patients whose initial EMRs listed dizziness as the chief complaint. The outcome was defined as the registration of dizziness-related diagnoses, including benign paroxysmal positional vertigo, sudden hearing loss, Ménière’s disease, vestibular neuritis, peripheral vertigo, cerebral infarction, and cerebral haemorrhage, or syncope-related diagnoses. Since the aim of this study is to inform clinical reasoning education, the emphasis is placed not on the methods for reaching a definitive diagnosis but on the reasoning process itself. Accordingly, the final diagnosis made by the physician, rather than the objectively accurate diagnosis, was considered the reference standard. The exclusion criteria were patients who were not registered with either a dizziness-related or syncope-related diagnosis at the first visit and patients from a department that did not treat both diseases. We focused only on data from departments that manage both diseases, as the study utilised a logistic regression model comparing two groups. This approach inevitably reduced the number of eligible patients. For example, in departments such as ophthalmology, patients are typically referred for only one of the two conditions, making it highly likely that the medical records reflect assumptions specific to that single diagnosis. The target departments for analysis were gastroenterology, nephrology, diabetology, respiratory medicine, allergy medicine, haematology, geriatrics, psychiatry, surgery, cardiovascular surgery, anaesthesiology, obstetrics and gynaecology, general medicine, and emergency medicine.

The second cohort comprised patients whose initial EMRs listed headaches as the chief complaint. The outcome was defined as the registration of neurosurgical diagnoses, including subarachnoid haemorrhage, cerebral haemorrhage, cerebral infarction, and brain tumour. As the outcome was the registration of neurosurgical diagnoses, patients without a neurosurgical consultation were excluded.

Text analysis of the initial EMRs was conducted using a Japanese natural language processing open-source library, GiNZA (GiNZA, version 5.1.2, Megagon Labs, https://github.com/megagonlabs/ginza, 2021, accessed on 17 June 2025), a package integrating morphological and dependency analyses [17]. In addition, we used Manbyo Dictionary Ver. 202106 (Manbyo-Dictionary, MANBYO_202106, Nara Institute of Science and Technology, https://sociocom.naist.jp/manbyou-dic/, 2021, accessed on 17 June 2025) to broadly extract symptom- and disease-related terms [18]. The extracted words were analysed using three steps: (1) calculating noun occurrence ratios for each outcome in binary classifications; (2) selecting words with more than a 10% difference in occurrence ratios between outcomes; (3) clinical selection by a physician. Moreover, the presence of affirmative or negative expressions for frequent terms was assessed based on GiNZA output for each patient’s initial EMR.

### Analysis

Logistic regression analyses were performed for the covariates, and variable selection was conducted based on the Akaike Information Criterion (AIC) [19]. The covariates were age, sex, and the presence or absence of words extracted in the previous three steps.

GiNZA analysis was conducted using Python 3.7.15, whereas logistic regression and decision tree analyses were performed using the glm function and rpart library in R version 4.1.3 (https://www.r-project.org/, accessed on 17 June 2025).

We conducted a multivariable logistic regression analysis to determine the odds ratios (ORs) and 95% confidence intervals of diagnoses. Independent variables were selected based on AIC criteria. Statistical significance was set at a two-tailed *p*-value of <0.05. All analyses were performed using R software (4.2.3).

## 3. Results

**Figure 1:**
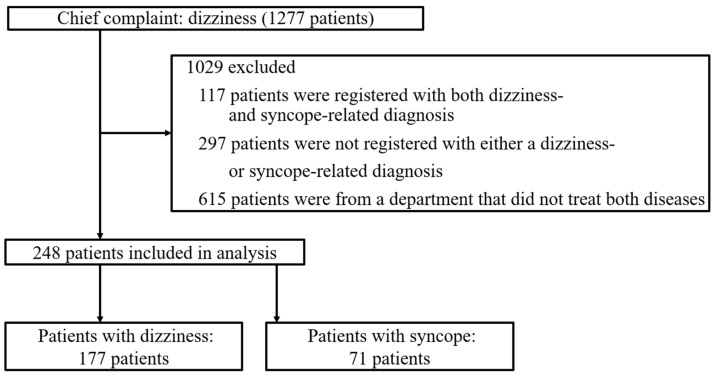
Inclusion of patients whose initial EMRs listed dizziness as the chief complaint.

We identified 1277 patients with dizziness as the chief complaint during the study period. Of these, 1029 met the exclusion criteria and 248 patients were included in the analysis (Figure 1). A total of 177 patients were diagnosed as related to vertigo, and 71 were diagnosed as related to syncope. The prior probability of a diagnosis of dizziness-related conditions was 71.3%. The analysis extracted 48 terms in three steps (Table 1).

The results of logistic regression analysis of the extracted terms without dependency relations are presented in Table 2. Terms with larger ORs were related to otolaryngology, including tinnitus (OR 113.056, 95% CI: 1166.102–10.961), vertigo (OR 94.618, 95% CI: 1171.896–7.639), otorhinolaryngology (OR 8.286, 95% CI: 46.983–1.462), and nystagmus (OR 5.966, 95% CI: 30.061–1.184). The area under the curve (AUC) for vertigo without dependency relations was 0.975, and the sensitivity, specificity, and F1-score were 0.944, 0930, and 0.957, respectively. ORs calculated by logistic regression analysis, incorporating affirmative and negative information along with age and sex, are presented in Table 3. The terms nystagmus (OR 4.25, 95% CI: 15.18–1.19), smoking (OR 0.16, 95% CI: 0.98–0.03), admission (OR 0.27, 95% CI: 0.85–0.09), vomiting (OR 4.48, 95% CI: 15.69–1.28), and examination (OR 0.14, 95% CI: 0.82–0.03) changed significant differences after adding dependency relations. The AUC for vertigo with dependency relations was 0.931, and the sensitivity, specificity, and F1-score were 0.876, 0.845, and 0.904, respectively. The AUC for vertigo with dependency relations was significantly lower than the AUC without dependency relations. (0.975 vs. 0.931, *p* = 0.001.)

**Figure 2:**
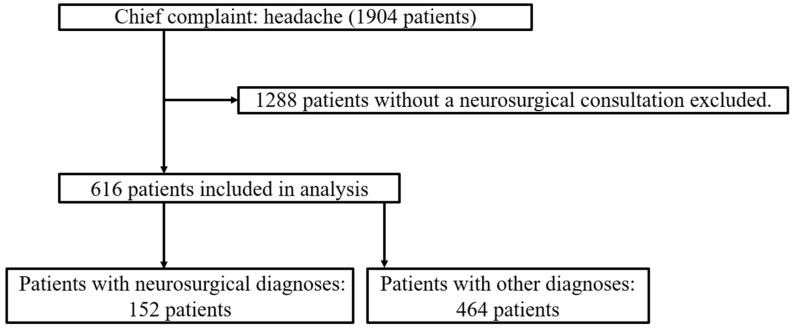
Inclusion of patients whose initial EMRs listed headache as the chief complaint.

We identified 1904 patients with headaches as their chief complaint during the study period. Of these, 616 patients evaluated by neurosurgeons were included in the analysis (Figure 2). A total of 152 patients had neurosurgical diagnoses and 464 had other diagnoses. The prior probability of neurosurgical diagnosis was 24.7%. The analysis extracted 45 terms in three steps (Table 4).

The results of the logistic regression analysis of the extracted terms are presented in Table 5. Terms with larger ORs were smoking (OR 28.44, 95% CI: 238.07–3.40), CT angiography (OR 19.76, 95% CI: 273.08–1.43), Japan Coma Scale (OR 15.48, 95% CI: 103.29–2.32), haematoma (OR 13.62, 95% CI: 35.97–5.16), and digital subtraction angiography (OR 12.54, 95% CI: 119.10–1.32). The AUC for vertigo without dependency relations was 0.916; the sensitivity, specificity, and F1-score were 0.763, 0.909, and 0.748, respectively. ORs calculated by logistic regression analysis, incorporating affirmative and negative information along with age and sex, are presented in Table 6. The terms CT (OR 1.91, 95% CI: 3.50–1.04), surgery (OR 5.86, 95% CI: 32.80–1.05), allergy (OR 5.41, 95% CI: 20.33–1.44), and subarachnoid haemorrhage (OR 5.64, 95% CI: 25.01–1.27) changed in significant differences after adding syntactic dependency information. The AUC for neurosurgical diagnoses with dependency relations were 0.923, and the sensitivity, specificity, and F1-score were 0.829, 0.879, and 0.754, respectively. The AUC for neurosurgical diagnoses with dependency relations was not significantly different from the AUC for neurosurgical diagnoses without dependency relations (0.916 vs. 0.923, *p* = 0.378).

## 4. Discussion

The results revealed that patients with documented vomiting were classified as experiencing dizziness. This finding aligns with clinical knowledge, as vomiting is common in both peripheral and central vertigo. Likewise, patients with a negative expression for nystagmus were classified as having dizziness. This suggests that nystagmus may have improved by the time of consultation, which is consistent with clinical experience in referred or chronic cases of dizziness.

Patients with a negative expression for smoking were classified as having syncope. Patients with suspected vascular conditions are frequently asked about smoking as part of their lifestyle history [20,21,22]. However, no established causal relationship exists between smoking and peripheral vertigo, which likely explains this finding.

Patients with documented hospitalisation were classified as having syncope. As hospitalisation occurred after the initial consultation, this finding suggests reverse causality, potentially reflecting the inclusion of patients with cardiogenic syncope.

Patients with a negative expression for tests were also classified as having syncope. If no abnormalities were found through diagnostic tests, the case may have been categorised as syncope. Clinical knowledge suggests that medical history is prioritised over tests for syncope evaluation, which is consistent with our findings [23].

Regarding the classification of neurosurgical conditions and others, the results identified the presence of computed tomography findings associated with neurosurgical conditions and affirmative expressions of subarachnoid haemorrhages indicating neurosurgical conditions. This was consistent with existing clinical knowledge. However, these associations may reflect reverse causality.

Negative expressions for surgery and allergy were associated with neurosurgical conditions. These findings may be related to standard enquiries made prior to surgical procedures, which could have introduced a diagnostic association.

In the dizziness cohort, the AUC for dizziness was significantly lower with the addition of dependency relations (AUC without dependency relations vs. with dependency relations: 0.975 vs. 0.931, *p* = 0.001). In the headache cohort, there was no significant difference in the AUC for neurosurgical diagnoses (AUC without dependency relations vs. with dependency relations: 0.916 vs. 0.923, *p* = 0.378). The addition of syntactic dependency information does not necessarily improve discrimination ability and may result in a slight decrease. However, the analysis revealed that the discrimination ability remained sufficient despite this decline. Furthermore, the number of significant affirmative and negative terms was minimal, indicating that the inclusion of dependency information allowed for better identification of word usage patterns. Therefore, validating dependency information is crucial for evaluating clinical reasoning. In addition, the model demonstrated high discriminative performance, with consistently high values for AUC, sensitivity, specificity, and F1-score. However, the primary aim of this study was to offer insights into clinical reasoning education. Accordingly, the emphasis was placed not on the process of arriving at a definitive diagnosis, but on the reasoning process itself. As such, the reference standard was the final diagnosis made by the physician, rather than an objectively confirmed diagnosis. It is therefore important to note that differences identified by the model may not necessarily correspond to clinically meaningful differences for accurate diagnosis.

EMRs offer opportunities to enhance medical education and improve interdisciplinary patient care [24]. Based on the results of this study, we documented the minimum set of keywords essential for clinical reasoning. However, terms commonly used to confine differential diagnoses, such as melena, dyspnoea, arrythmia, and chest pain, have rarely been used in this study [25,26,27]. Using EMR documentation as an educational tool may be useful for diagnostic purposes but may be less effective for exclusionary reasoning. Specific differential diagnosis terms could have been absent because the study population consisted of patients who visited a university hospital. University hospitals often handle referred patients treated by specialists, who may omit self-evident details from their documentation.

The methodology employed was not complex. Text analysis was performed using a Japanese natural language processing open-source library to extract terms, classify them based on frequency, incorporate dependency information, and subsequently perform logistic regression analysis. Artificial intelligence applications have advantages in diagnostic imaging and treatment selection [16,28,29]. However, there are limitations to making diagnostic inferences from words in electronic medical records. Deep learning and artificial intelligence are complex processes. The present method is classical and simple and allows for a clear reasoning process. Therefore, it can be applied in teaching diagnostic reasoning to residents and medical students.

In this study, we employed only logistic regression analysis. Our primary objective was not to achieve accurate diagnostic predictions, but rather to evaluate the influence of individual words on diagnostic outcomes. Although decision trees could also be used to assess the impact of word presence or absence, we chose logistic regression because it is the most conventional method and offers straightforward interpretability. While we are interested in using more advanced models—such as hierarchical models—to examine the relationships between words, the current sample size is insufficient for such approaches. Nevertheless, our findings demonstrate that even a simple logistic regression model can be used effectively to assess the influence of specific words on diagnoses using electronic medical records.

Clinicians use EMRs to systematically organise patient information and construct cognitive pathways during documentation and review processes. These pathways facilitate clinical reasoning, enabling informed decision-making regarding diagnosis and treatment planning [15,30]. This study aimed to evaluate the impact of individual words in initial EMRs on diagnostic outcomes. A high OR for a term suggested that it was commonly documented by clinicians when considering a particular diagnosis, indicating its importance in confirming the diagnosis. Therefore, this method provides fundamental data to assess reasoning processes, suggesting that even simple techniques such as logistic regression analysis, rather than advanced technologies such as machine learning, are effective in evaluating human cognitive processes.

### Limitations

This study had several limitations. First, it was a single-centre study conducted at a university hospital, which may have introduced a data bias due to the high proportion of patients referred from other institutions. Furthermore, the specialty of the physicians who recorded the data was not taken into consideration, and it is possible that the records were made by specialists. Nevertheless, considering the scarcity of studies on this topic, these findings hold significance. Regarding transferability, the methods used in this study are relatively simple, and therefore, with a sufficient sample size, similar analyses could be feasibly conducted at other institutions. In the future, this method could be applied to research in general hospitals and to differences in EMRs according to specialisation. Second, EMRs may not have included all of the information. Information that could be risky to the patient, such as information of patients with post-traumatic stress disorder, is usually not included in the chart [31]. However, the words headache and dizziness are unlikely to pose a risk to the patient; thus, including them would be acceptable. Third, there is a potential for selection bias. Because the present study employed a logistic regression model to compare two diagnostic groups, the analysis was limited to data from departments that manage both conditions. This approach inevitably reduced the number of eligible patients and may have introduced dependence on documentation practices specific to certain departments. Furthermore, the characteristics of patients in whom both conditions were suspected were not considered. Finally, the words included in the logistic regression analysis were selected based on differences in frequency of occurrence between groups. This selection criterion may have excluded terms that should have been documented in both groups during the diagnosis. In addition, the selection of terms based solely on frequency differences may have excluded rare but clinically significant terms. Given the exploratory nature of this study and its primary aim to identify differences between groups, the evaluation of terms based solely on frequency differences was deemed sufficient. Future studies may ensure that critical words are always included.

## 5. Conclusions

This study demonstrated that initial EMRs contained patient background information relevant to diagnosis. Combining this information with diagnostic test data could enhance the assessment of the clinical reasoning process. In addition, the methods used in this study could be implemented in further research to address biases in EMR data, optimise EMR design, and develop interdisciplinary collaboration, thereby improving healthcare delivery.

## Figures and Tables

**Table 1:** List of 48 terms extracted from cohort 1 (dizziness).

Extracted Terms		
Referral	Nystagmus	Otolaryngology
Vertigo	Admission	Medication
Medical checkup	Vomiting	ECG (Electrocardiogram)
Head	Finger	Past medical history
MRI	Chest	Anaemia
Appetite	ALT (Alanine Aminotransferase)	Body weight
This study hospital	Eyes	Gait
HB (Haemoglobin)	Ultrasound	CRP (C-Reactive Protein)
Tinnitus	Alcohol consumption	Smoking
HR (Heart Rate)	WBC (White Blood Cell count)	Platelets (Plt)
Palpitations	Fatigue	Prescribed medications
Limbs	Further examination	RBC (Red Blood Cell count)
Nose	Blood test	Hearing loss
Surgery	AST(Aspartate Aminotransferase)	Laboratory test
Headache	Family	Asthma
Family history	Symptom	Outpatient visit

**Table 2:** Logistic regression analysis results for vertigo without dependency relations.

Variable	Odds Ratio	*p*-Value
Male	3.022 (12.031–0.759)	0.117
Age	1.033 (1.069–0.999)	0.057
Referral	0.254 (0.858–0.075)	0.027
Tinnitus	113.056 (1166.102–10.961)	<0.001
Nystagmus	5.966 (30.061–1.184)	0.030
Alcohol consumption	0.336 (1.374–0.082)	0.129
Otorhinolaryngology	8.286 (46.983–1.462)	0.017
Hb	0.054 (0.302–0.010)	<0.001
Vertigo	94.618 (1171.896–7.639)	<0.001
Heart rate	0.015 (0.117–0.002)	<0.001
Admission	0.362 (1.429–0.092)	0.147
Family history	0.004 (0.516–0.000)	0.026
Palpitation	0.007 (0.089–0.001)	<0.001
Vomiting	5.753 (30.490–1.085)	0.040
Fatigue	0.053 (0.478–0.006)	0.009
Ultrasound	0.068 (0.548–0.008)	0.012
Limbs	9.762 (60.713–1.570)	0.015
Finger	5.970 (24.344–1.464)	0.013
Symptom	8.336 (34.470–2.016)	0.003
Chest	3.333 (17.749–0.626)	0.158
Blood	0.175 (0.863–0.036)	0.032
Body weight	4.904 (35.341–0.681)	0.115
The study hospital	0.164 (0.747–0.036)	0.019
Family	0.222 (1.146–0.043)	0.072

The area under the curve was 0.975, the sensitivity was 0.944, the specificity was 0.930, and the F1-score was 0.957.

**Table 3:** Logistic regression analysis results for vertigo with dependency relations.

Variable	Odds Ratio	*p*-Value
Male	4.57 (14.88–1.40)	0.012
Age	1.02 (1.05–0.99)	0.152
Referral (+)	0.27 (0.71–0.10)	0.008
Tinnitus (+)	16.35 (114.45–2.34)	0.005
Nystagmus (−) *	4.25 (15.18–1.19)	0.026
Alcohol consumption (+)	0.23 (1.08–0.05)	0.062
Otorhinolaryngology (+)	6.73 (22.36–2.02)	0.002
Smoking (+) *	0.16 (0.98–0.03)	0.047
Heart rate (+)	0.23 (0.79–0.07)	0.019
Admission (+) *	0.27 (0.85–0.09)	0.025
Plate (+)	0.09 (0.48–0.02)	0.005
Palpitation (+)	0.08 (0.37–0.02)	0.001
Vomiting (+) *	4.48 (15.69–1.28)	0.019
Vomiting (−)	4.38 (34.24–0.56)	0.159
Headache (+)	3.95 (13.48–1.16)	0.028
Limbs (−)	5.60 (34.01–0.92)	0.061
Symptom (−)	2.77 (8.62–0.89)	0.079
Chest (−)	0.24 (1.30–0.04)	0.097
Blood (+)	0.07 (0.30–0.02)	<0.001
Anaemia (−)	0.38 (1.34–0.11)	0.133
Appetite (+)	4.46 (15.57–1.28)	0.019
Examination (−) *	0.14 (0.82–0.03)	0.029
Headache (−)	5.29 (30.37–0.92)	0.062
Family (+)	0.16 (0.63–0.04)	0.008

The area under the curve was 0.931 (*p* = 0.001), the sensitivity was 0.876, the specificity was 0.845, and the F1-score was 0.904. (+), variables with affirmative expressions; (−), variables with negative expressions. * Variables with changes in significant differences after adding syntactic dependency information.

**Table 4:** List of 45 terms extracted from cohort 2 (headaches).

Extracted Terms		
Referral	Medication	Cerebrospinal fluid leakage
JCS (Japan Coma Scale)	Hypertension	Smoking
Aneurysm	Treatment	MRI
Transport	Internal medicine	Neurology
Dysarthria	Sensory disorder	Haematoma
Family history	Blood pressure	Dizziness
Artery	SAH (Subarachnoid Haemorrhage)	Disturbance of consciousness
Migraine	Alcohol consumption	Emergency
Admission	Surgery	Observation
Test	Visual field	Sensory disturbance
Allergy	ECG	Cerebral infarction
DSA (Digital Subtraction Angiography)	NIHSS (National Institutes of Health Stroke Scale)	MRA (Magnetic Resonance Angiography)
Vomiting	Ventricle	CT
CTA (CT Angiography)	Facial palsy	Neurological findings
Aphasia	Tension headache	Oculomotor dysfunction

**Table 5:** Logistic regression analysis results for headache without dependency relations.

Variable	Odds Ratio	*p*-Value
Age	1.01 (1.03–1.00)	0.051
Referral	3.10 (5.73–1.67)	<0.001
Admission	2.42 (5.14–1.14)	0.022
CT	1.62 (2.88–0.91)	0.099
Smoking	28.44 (238.07–3.40)	0.002
JCS (Japan Coma Scale)	15.48 (103.29–2.32)	0.005
Test	1.87 (3.84–0.91)	0.089
Surgery	1.79 (3.65–0.87)	0.112
MRI	1.73 (3.12–0.96)	0.068
Sensory disorder	0.39 (1.31–0.11)	0.127
Transport	2.74 (9.13–0.83)	0.100
DSA (Digital Subtraction Angiography)	12.54 (119.10–1.32)	0.028
Internal Medicine	1.84 (4.17–0.81)	0.144
Ventricle	3.95 (15.09–1.03)	0.045
Haematoma	13.62 (35.97–5.16)	<0.001
Subarachnoid haemorrhage	3.56 (14.62–0.87)	0.078
Facial palsy	2.72 (9.02–0.82)	0.102
Dizziness	2.06 (5.16–0.82)	0.122
Artery	2.53 (5.46–1.17)	0.018
CTA (CT Angiography)	19.76 (273.08–1.43)	0.026
Tension headache	0.13 (1.03–0.02)	0.053
Cerebrospinal fluid leakage	0.14 (0.68–0.03)	0.015

The area under the curve was 0.916, the sensitivity was 0.763, the specificity was 0.909, and the F1-score was 0.748.

**Table 6:** Logistic regression analysis results for headache with dependency relations.

Variable	Odds Ratio	*p*-Value
Age	1.01 (1.03–1.00)	0.100
Referral (+)	3.28 (6.14–1.75)	<0.001
Admission (+)	2.50 (5.49–1.14)	0.023
CT (+) *	1.91 (3.50–1.04)	0.036
Smoking (+)	19.16 (243.42–1.51)	0.023
JCS (+)	15.04 (88.83–2.55)	0.003
Test (+)	1.91 (3.98–0.92)	0.082
Internal Medicine (+)	1.74 (3.71–0.82)	0.152
Surgery (−) *	5.86 (32.80–1.05)	0.044
Observation (+)	0.51 (1.08–0.24)	0.080
Allergy (−) *	5.41 (20.33–1.44)	0.012
SAH (+) *	5.64 (25.01–1.27)	0.023
Neurology (−)	0.48 (1.33–0.17)	0.159
DSA (+)	6.86 (64.34–0.73)	0.092
MRA (+)	2.90 (7.67–1.10)	0.032
Ventricle (+)	4.91 (18.64–1.30)	0.045
Haematoma (+)	18.13 (54.01–6.09)	<0.001
Shoulder pain (+)	0.38 (1.51–0.10)	0.170
Dizziness (+)	2.73 (7.45–1.00)	0.049
Neurological findings (−)	0.50 (1.28–0.19)	0.150
Artery (+)	3.75 (9.59–1.46)	0.006
Tension headache (+)	0.11 (1.26–0.01)	0.077
CSF leakage (+)	0.18 (0.98–0.03)	0.048

The area under the curve was 0.923 (*p* = 0.378), the sensitivity was 0.829, the specificity was 0.879, and the F1-score was 0.754. (+), variables with affirmative expressions; (−), variables with negative expressions. * Variables with changes in significant differences after adding syntactic dependency information.

## Data Availability

The datasets used or analysed in this study are available from the corresponding author upon reasonable request and with permission from the Ethical Review Committee of Kochi University School of Medicine.

## Abbreviations

The following abbreviations are used in this manuscript:

EMR	electronic medical record
AIC	Akaike Information Criterion
OR	odds ratio
AUC	area under the curve
